# HIV and Syphilis Co-Infection Increasing among Men Who Have Sex with Men in China: A Systematic Review and Meta-Analysis

**DOI:** 10.1371/journal.pone.0022768

**Published:** 2011-08-15

**Authors:** Eric P. F. Chow, David P. Wilson, Lei Zhang

**Affiliations:** The Kirby Institute for Infection and Immunity in Society, Faculty of Medicine, University of New South Wales, Sydney, Australia; UCL Institute of Child Health, University College London, United Kingdom

## Abstract

**Background:**

This study aims to estimate the magnitude and changing trends of HIV, syphilis and HIV-syphilis co-infections among men who have sex with men (MSM) in China during 2003–2008 through a systematic review of published literature.

**Methodology/Principal Findings:**

Chinese and English literatures were searched for studies reporting HIV and syphilis prevalence among MSM from 2003 to 2008. The prevalence estimates were summarized and analysed by meta-analyses. Meta-regression was used to identify the potential factors that are associated with high heterogeneities in meta-analysis. Seventy-one eligible articles were selected in this review (17 in English and 54 in Chinese). Nationally, HIV prevalence among MSM increased from 1.3% during 2003–2004 to 2.4% during 2005–2006 and to 4.7% during 2007–2008. Syphilis prevalence increased from 6.8% during 2003–2004 to 10.4% during 2005–2006 and to 13.5% during 2007–2008. HIV-syphilis co-infection increased from 1.4% during 2005–2006 to 2.7% during 2007–2008. Study locations and study period are the two major contributors of heterogeneities of both HIV and syphilis prevalence among Chinese MSM.

**Conclusions/Significance:**

There have been significant increases in HIV and syphilis prevalence among MSM in China. Scale-up of HIV and syphilis screening and implementation of effective public health intervention programs should target MSM to prevent further spread of HIV and syphilis infection.

## Introduction

Over the last decade in many international settings there have been resurgences in syphilis epidemics among men who have sex with men (MSM), which have been strongly associated with the increases in HIV incidence [1]–[5]. Until relatively recently, syphilis has been well-controlled in much of the world, including China; syphilis was nearly eradicated in China in 1964 [6]. Syphilis infection has now returned to become one of the top five most reported notifiable diseases in China [7]–[10]. There was a 30-fold increase in syphilis diagnoses in China from 0.2 cases per 100,000 individuals in 1989 to 6.5 cases per 100,000 individuals in 1999 and a further 3-fold increase over the following decade to 22 cases per 100,000 individuals in 2008 [11]–[13]. The estimated number of people living with HIV in China has also increased markedly from 400,000 in 2007 to 740,000 in 2009 [14]. In 2009, 32.5% of new HIV infections was attributed to male homosexual exposure, which is almost triple the reported level in 2007 of 12.2% [15].

Sexual exposure has become the primary route of HIV transmission in China in recent years [16] and is the mode of transmission for syphilis infection. There are statistical associations between syphilis infection and HIV acquisition, and due to biologically plausible reasons the presence of such a sexually transmissible infection can facilitate HIV transmission [17], [18]. Furthermore, due to a higher transmission probability of HIV associated with penile-anal intercourse [19] and levels of risk-related behaviour, Chinese MSM have an approximately 45-fold higher risk of acquiring HIV than other males in the general population [20]. It is therefore important to understand current prevalence levels of HIV, syphilis and HIV-syphilis co-infection among MSM in China. There have been numerous individual studies that have separately reported the prevalence of HIV or syphilis among MSM in a selected sample population from specific urban cities in China at a specific time, but very few studies have reported on co-infection [21]–[33]. Across these studies, large variations in HIV and syphilis prevalence estimates are commonly observed, reflecting heterogeneous geographical and chronological changes as both epidemics have evolved. Therefore, in this study we investigate the temporal trends and geographical patterns of these epidemics and their likely interaction through a systematic review. We review, update and summarize the current magnitude and patterns of HIV and syphilis epidemics, as well as their co-infection epidemic among MSM in China.

## Methods

### Search strategy

Two independent investigators (EPFC, LZ) conducted a systematic review of published peer-reviewed research articles by searching the following databases: PubMed, Chinese Scientific Journals Fulltext Database (CQVIP), China National Knowledge Infrastructure (CNKI) and Wanfang Data from 2003–2010. Keywords used in the database search included (“HIV” *OR* “AIDS”) *OR* (“STD” *OR* “Sexually transmitted diseases” *OR* “Syphilis”) *OR* (“co-infection”) *AND* (“homosexual” *OR* “gay” *OR* “bisexual” *OR* “men who have sex with men” OR “MSM”) *AND* “China”. We also performed a manual search on the reference lists of published articles. This review was conducted and reported according to the PRISMA (Preferred Reporting Items for Systematic Reviews and Meta-Analyses) Statement issued in 2009 (Table S1) [34].

### Study selection

Studies were eligible for inclusion in this systematic review if they met the following criteria: (1) study published in Chinese or English language; (2) study reported both HIV and syphilis prevalence estimates among MSM in China; (3) HIV and syphilis infection must be diagnosed from laboratory serologic testing; (4) study design such as study site, time period and sample size must be reported. The term ‘MSM’ also included male sex workers (‘money boys’). To avoid overestimation of the HIV and syphilis prevalence among the general MSM population, studies with small proportions of ‘money boys’ (less than 10% of the total sample) were also included in this study.

We excluded review papers, non peer–reviewed local/government reports, conference abstract and presentation in this study. Self-reported HIV or syphilis infections were excluded. If the same study data were published in both English and Chinese sources, the articles published in Chinese language were excluded from the review.

Non-treponemal tests are non-virus-specific tests that are often used in syphilis screening, whereas virus-specific treponemal tests are necessary for syphilis infection confirmation. Positive results from both treponemal and non-treponemal syphilis tests confirm a current infection. In this review, we investigated the temporal prevalence of current syphilis infection. Studies with unknown syphilis testing methods or missing one of the non-treponemal and treponemal syphilis tests were further excluded in quantitative synthesis (meta-analysis).

### Validity assessment

Studies were considered higher quality by the following criteria: (1) studies used both non- treponemal and treponemal test for syphilis diagnosis; (2) studies reported HIV-syphilis co-infection; (3) cohort studies; (4) sample size of studies were greater than 200; (5) two or more study sites and targeted methods were used for MSM recruitment; and (6) studies published in English.

### Data abstraction

We extracted the following information from all eligible studies: first author and published year; study site; age of MSM participants; study period; study base and method; sample size; testing method for syphilis; prevalence of HIV; prevalence of syphilis; prevalence of HIV-syphilis co-infection; The studies were categorized by geographical location, according to the six traditional Chinese regions of administrative division (East China, Northeast China, North China, South Central China, Northwest China and Southwest China) and presented in Table S2. In addition, based on availability of data, the studies were further categorised into three two-year time periods: 2003–2004, 2005–2006 and 2007–2008 (Table S2).

### Statistical Analysis

Meta-analyses were carried out using the Comprehensive Meta-Analysis software (V2.0, Biostat, Englewood, New Jersey). The effect rates of pooled prevalence estimates and 95% confidence intervals (CI) for each study were determined by using random effect models. Heterogeneity tests were performed using the Cochran Q-test (*p*<0.10 represents statistically significant heterogeneity) and *I* ^2^ statistic. We investigated the factors that are associated with heterogeneities in the stratified meta-analyses using meta-regression analysis. Potential presence of publication bias was measured by the Begg and Mazumdar rank correlation. Spearmen correlation was used to assess the relationship between HIV prevalence and syphilis prevalence among MSM in China.

## Results

### Trial Flow/Flow of included studies

**Figure 1:**
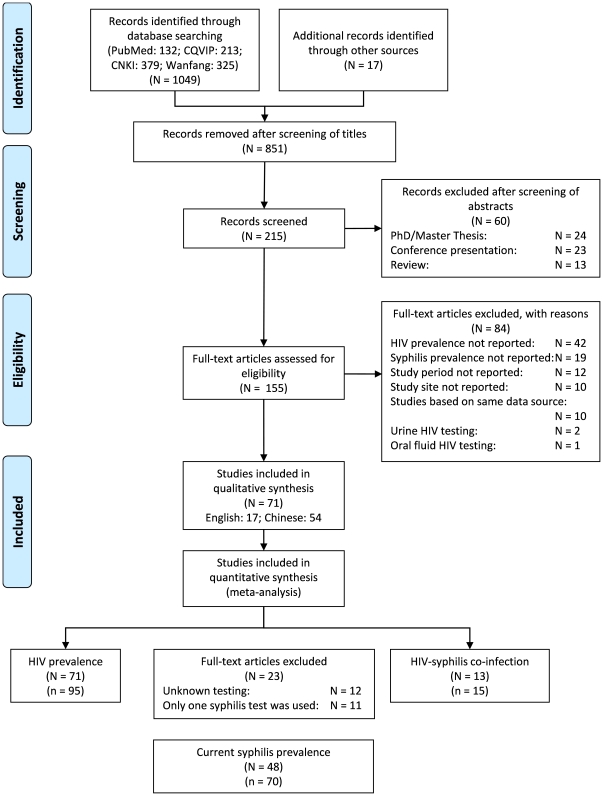
Flow chart showing the meta-analysis studies selection. N; the number of articles included in systematic review; n, the number of prevalence estimates included in meta-analysis.

Our initial search criteria identified 1049 articles from four electronic databases and 17 additional articles were identified through reference lists from identified articles. We excluded 851 articles because they were unrelated to the topics or duplicated titles from different databases. Abstracts were screened among the remaining 215 articles and 60 were excluded because they were student theses (N = 24), conference presentations (N = 23) and review papers (N = 13). One-hundred and fifty-five articles were eligible for full-text screening and 84 were further excluded as they did not report HIV prevalence (N = 30), syphilis prevalence (N = 19), study period (N = 12), study site (N = 10), studies based on the same data source (N = 10), or did not use blood sample for HIV diagnosis (N = 3). The remaining 71 studies (17 in English and 54 in Chinese) were eligible in qualitative synthesis. We performed stratified meta-analyses for HIV, syphilis and HIV-syphilis prevalence estimates. We included all the eligible studies for HIV prevalence in meta-analysis (71 articles, 95 HIV prevalence estimates) [21]–[92]. We excluded 23 articles for syphilis prevalence because among which 12 did not report the syphilis testing method and 11 used either treponemal or non-treponemal syphilis test only. The remaining 48 articles (70 syphilis prevalence estimates) were eligible in meta-analysis for current syphilis prevalence estimates [21]–[79]. Fourteen articles reported HIV-syphilis co-infection prevalence estimates but one was excluded because only one syphilis test was used [74]. The remaining 13 articles (15 HIV-syphilis co-infection prevalence estimates) were included in the meta-analysis [21]–[33]. The selection process is illustrated in Figure 1.

### Study characteristics

Out of 31 Chinese provinces, the eligible articles included in this study covered 24 provinces (no studies indentified in Tibet, Qinghai, Henan, Hunan, Jingxi, Fujian and Shanxi provinces). In the total 71 studies, the sample size of the selected studies ranged from 19 to 1692 (median: 296; 95% CI: 137–456); 31 studies (51%) recruited MSM participates from MSM venues but 7 studies (10%) did not report the study base. Thirty-five out of 71 studies (49%) did not report the study method, 18 studies (25%) used snowball method and 12 studies (17%) used respondent-driven sampling (RDS) method. There were 9, 36 and 50 HIV prevalence estimates in the time periods 2003–2004, 2005–2006 and 2007–2008, respectively; while the number of syphilis prevalence estimates was 4, 26 and 40. There were 7 and 8 HIV-syphilis co-infection prevalence estimates reported in the period 2005–2006 and 2007–2008, respectively, no co-infection prevalence was reported during 2003–2004.

### Epidemic trends of HIV, syphilis infection and HIV-syphilis co-infection

**Figure 2:**
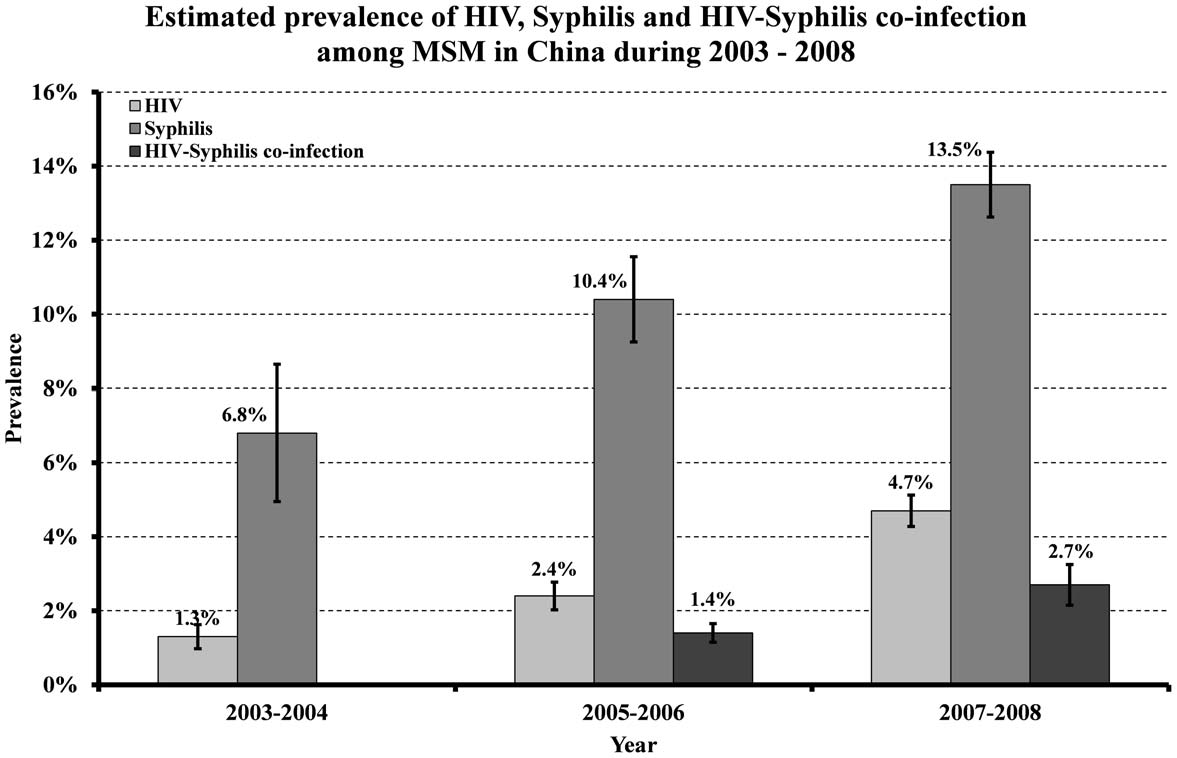
Estimated prevalence of HIV, syphilis infection and HIV-syphilis co-infection among men who have sex with men during 2003–2008. Each column represents the pooled estimate from meta-analyses over all studies for the given region and time period. The error bars represent standard deviation of the percentages.

**Figure 3:**
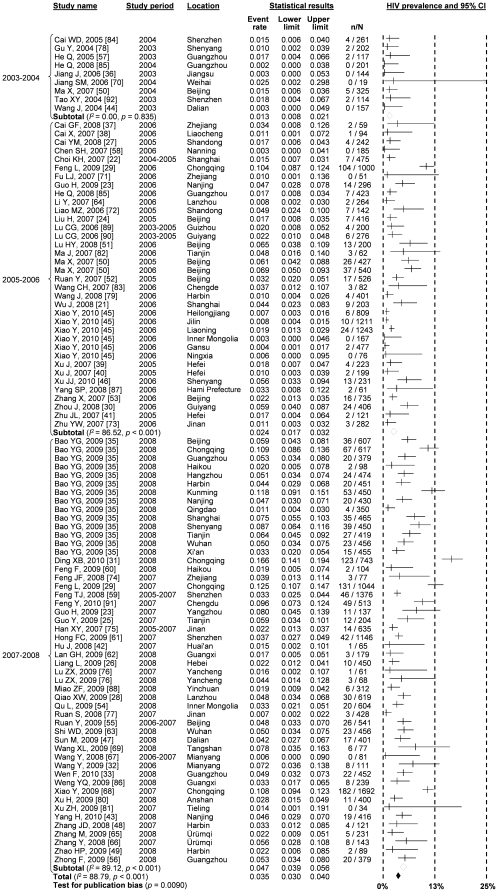
Forest Plot showing the results of meta-analyses of HIV prevalence among MSM in different time period.

According to our pooled prevalence estimates from meta-analyses across all grouped studies, the overall national HIV prevalence among MSM in China has increased substantially from 1.3% (95% CI: 0.8–2.1%) during 2003–2004 to 2.4% (95% CI: 1.7–3.2%) during 2005–2006 and then to 4.7% (95% CI: 3.9–5.6%) during 2007–2008 (Figure 2, 3). A significant increasing trend was found by linear regression, which estimated a 0.9% (95% CI: 0.5–1.3%, *p*<0.001) increase in HIV prevalence among Chinese MSM across the time periods. Significant publication bias (*p* = 0.009) and high heterogeneity (*I* ^2^ = 88.79; Q test *p*<0.0001) were observed. Meta-regression analysis showed that this high heterogeneity was significant associated with the geographical locations of the studies (*β* = 0.104, *p* = 0.024) and study time period (*β* = 0.236, *p* = 0.001) (Table 1).

**Table 1:** Result of individual variable meta-regression models for each stratified meta-analysis.

			Stratified meta-analyses			
Study Characteristics	HIV		Syphilis		HIV-syphilis co-infection	
	Prevalence (95% CI)	Meta-regression (β, *p*-value)	Prevalence (95% CI)	Meta-regression (β, *p*-value)	Prevalence (95% CI)	Meta-regression (β, *p*-value)
*Language of article:*						
Chinese	3.8% (3.1–4.5%)	−0.051	11.5% (10.0–13.2%)	**0.320**	1.8% (1.2–2.9%)	0.349
English	2.5% (1.7–3.7%)	*p* = 0.793	12.7% (10.4–15.5%)	***p*** ** = 0.059**	2.3% (1.4–4.0%)	*p* = 0.559
*Sample size:*						
<200	3.1% (2.3–4.0%)	0.098	11.8% (8.9–15.4%)	−0.191	5.0% (2.8–8.5%)	−0.521
≥200	3.6% (3.0–4.4%)	*p* = 0.623	11.8% (10.4–13.4%)	*p* = 0.272	1.8% (1.6–2.6%)	*p* = 0.429
*Study base:*						
MSM venues	3.0% (2.3–3.9%)	0.068	11.6% (9.7–13.8%)	0.043	2.2% (1.5–3.1%)	−0.516
Non MSM venues	3.0% (2.2–4.2%)	*p* = 0.522	12.3% (10.5–14.3%)	*p* = 0.612	1.6% (0.7–3.8*)	*p* = 0.393
*Sampling method:*						
RDS/snowball	3.4% (2.7–4.2%)	0.121	11.4% (9.9–13.1%)	−0.078	2.2% (1.6–3.1%)	−0.217
Others	4.7% (2.7–8.1%)	*p* = 0.386	9.1% (8.0–10.3%)	*p* = 0.308	1.6% (0.7–3.9%)	*p* = 0.548
*Study locations* * *:*						
East	3.0% (2.2–4.0%)		11.8% (9.2–15.0%)		2.5% (1.1–5.6%)	
Northeast	2.3% (1.4–3.8%)		13.7% (10.1–18.3%)		N/A	
North	4.2% (3.3–5.3%)	**0.104**	15.0% (12.0–18.6%)	**−0.086**	1.1% (0.3–4.2%)	−0.041
South Central	3.4% (2.7–4.2%)	***p*** ** = 0.024**	13.9% (11.3–16.9%)	***p*** ** = 0.036**	1.6% (1.1–2.5%)	*p* = 0.860
Northwest	2.5% (1.5–4.0%)		6.7% (3.8–11.4%)		N/A	
Southwest	9.2% (7.4–11.2%)		8.2% (6.8–9.8%)		2.5% (1.5–4.0%)	
*Time period:*						
2003–2004	1.3% (0.8–2.1%)	**0.236**	6.8% (4.0–11.4%)	**0.194**	N/A	0.479
2005–2006	2.4% (1.7–3.2%)	***p*** ** = 0.001**	10.4% (8.3–12.9%)	***p*** ** = 0.002**	1.4% (0.8–2.3%)	*p* = 0.090
2007–2008	4.7% (3.9–5.6%)		13.5% (11.8–15.3%)		2.7% (1.8–4.0%)	

*Study locations were categorized into six Chinese traditional regions. East China: Anhui, Fujian, Jiangsu, Jiangxi, Shandong, Shanghai, Zhejiang; Northeast China: Heilongjiang, Jilin, Liaoning; North China: Beijing, Hebei, Inner Mongolia, Shanxi, Tianjin; South Central China: Guangdong, Guangxi, Hainan, Henan, Hubei, Hunan; Northwest China: Gansu, Ningxia, Qinghai, Shaanxi, Xinjiang; Southwest China: Chongqing, Guizhou, Sichuan, Tibet, Yunnan.

The meta-regression coefficient (β) and the significant of β (*p* value) for each study characteristic were reported.

**Figure 4:**
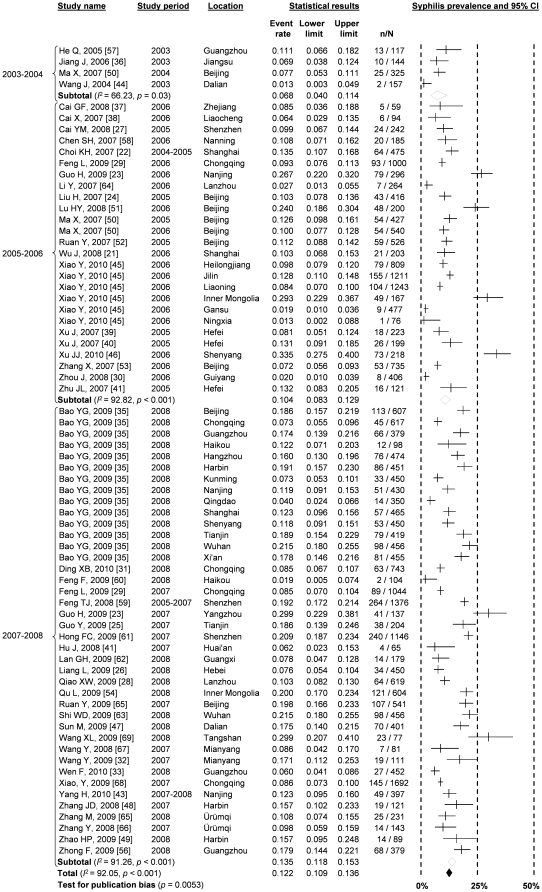
Forest Plot showing the results of meta-analyses of syphilis prevalence among MSM in different time period.

Prevalence of syphilis also increased substantially among Chinese MSM during the studied time periods. It increased from 6.8% (95% CI: 4.0–11.4%) during 2003–2004 to 10.4% (95% CI: 8.3–12.9%) during 2005–2006 and then to 13.5% (95% CI: 11.8–15.3%) during 2007–2008 (Figure 2, 4). This corresponds to a marginal significant increase of 1.2% (95% CI: −0.04–2.5%, *p* = 0.05) among Chinese MSM. The analysis demonstrated high heterogeneity (*I* ^2^ = 92.05; Q test *p*<0.001) which is significantly associated with the language of articles (*β* = 0.320, *p* = 0.059), study location (*β* = −0.086, *p* = 0.036) and study time period (*β* = 0.194, *p* = 0.002) (Table 1). Significant publication bias (*p* = 0.005 for Begg rank correlation analysis) was observed.

**Figure 5:**
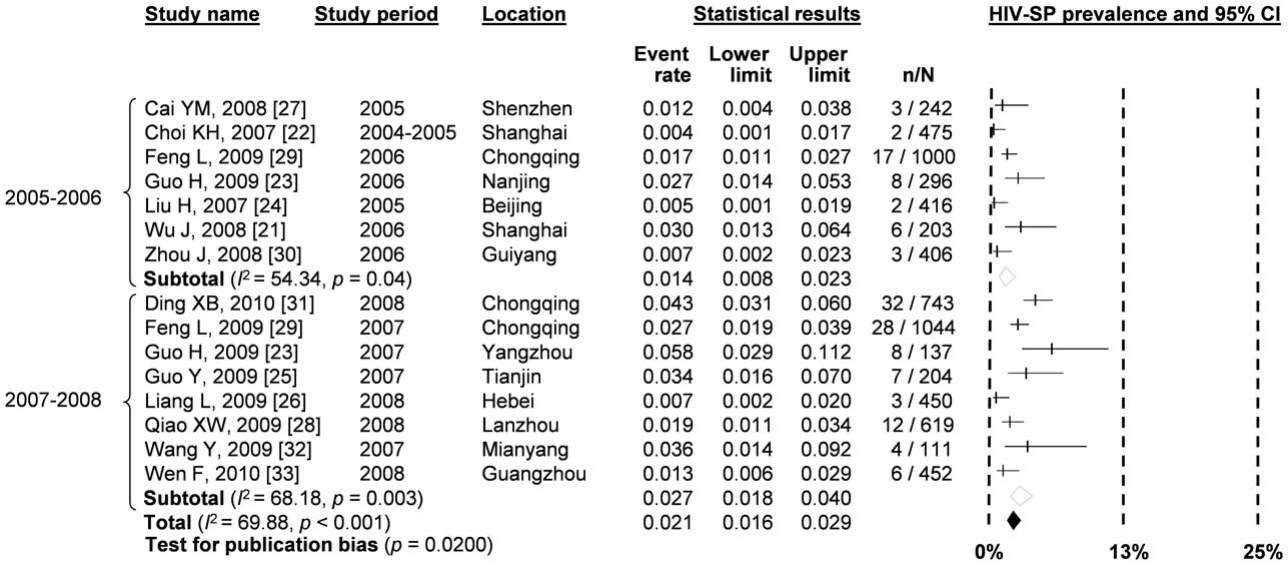
Forest Plot showing the results of meta-analyses of HIV-syphilis co-infection prevalence among MSM in different time period.

Pooled prevalence estimates of HIV-syphilis co-infection across the country substantially increased from 1.4% (95% CI: 0.8–2.3%) during 2005–2006 to 2.7% (95% CI: 1.8–4.0%) during 2007–2008 (Figure 2, 5), with an absolute increase of 0.5% (95% CI: −0.2–1.3%). However, the increasing trend was not statistically significant (*p* = 0.16). A significantly high level of heterogeneity was found between studies (*I* ^2^ = 69.88; Q test *p*<0.001) and publication bias was also observed (*p* = 0.02 for Begg rank correlation analysis). None of the study characteristics were significantly associated with the presence of high heterogeneity (Table 1).

### Association between HIV and syphilis infection

**Figure 6:**
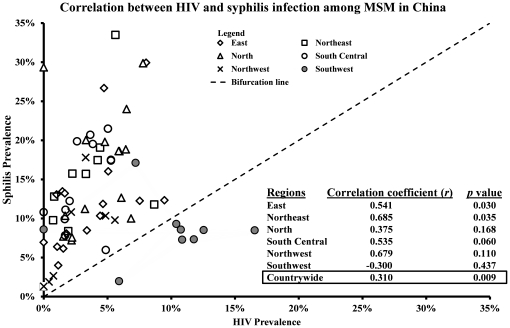
Correlation between HIV and Syphilis infection among men who have sex with men in China.

A significantly positive correlation (*r* = 0.31, *p* = 0.009) was observed between HIV and syphilis prevalence among Chinese MSM during 2003–2008 countrywide (Figure 6). Significant positive correlation was also observed in the East region (*r* = 0.54, *p* = 0.030) and the Northeast region (*r* = 0.69, *p* = 0.035) and marginally significant positive correlation in the South central region (*r* = 0.54, *p* = 0.060). Interestingly, in almost all regions syphilis prevalence was found to be higher than HIV prevalence. The only exception was observed in the Southwest region with more studies reporting HIV prevalence than syphilis prevalence.

## Discussion

Our review showed that epidemics of HIV, syphilis and HIV-syphilis co-infection among MSM in China has substantially increased from 2003 to 2008. The HIV, syphilis and co-infection prevalence increased at an annual rate of 0.9% (95% CI: 0.5–1.3%), 1.2% (95% CI: −0.04–2.5%) and 0.5% (95% CI: −0.2–1.3%) respectively during the studied period. In general, syphilis prevalence is much higher than HIV prevalence among MSM across China. To our knowledge this is the first time that co-infection of HIV and syphilis among MSM in China was reviewed over time by meta-analysis.

Several limitations in this study should be noted. Our estimated syphilis prevalence of 6.8% during 2003–2004 was based on only four prevalence estimates and there was considerable variation across the studies. Even in other time periods of our analysis, the prevalence data of HIV and syphilis were collected in large urban cities in China and hence likely to be over-estimates of prevalence levels across the entire provinces, including rural areas. Further investigation among MSM in rural areas and smaller cities in urban areas are necessary to provide a more accurate description of HIV and syphilis epidemics. Significant publication biases were observed in the stratified meta-analyses of HIV, syphilis and HIV-syphilis prevalence among Chinese MSM. The selected studies had a large range of sample sizes (19 to 1962). Researchers are more likely to publish large studies because small studies have less statistical power in comparison with large studies [93]. Our study targeted for HIV and syphilis infections among the same study cohorts, studies reporting only HIV or syphilis prevalence estimates were excluded in this review and hence some significant studies might be missing in the stratified meta-analyses, which caused the presence of publication bias. There is likely to be other publication bias due to our strict inclusion criteria. Researchers are more likely to publish and report high HIV and syphilis prevalence [93], [94]. There are also a potentially large number of governmental documents, community-level NGO reports and other unpublished data that have never been archived in any of the public literature databases. Our strict inclusion criteria of the presence of both treponomal and non-treponomal test for syphilis infection may also be a source of bias. Over one-third of syphilis studies (23/71) did not report testing methods or both tests for syphilis were consequently excluded (Table S2).

The presence of high heterogeneities was also an important limitation in this study. These heterogeneities might be attributable to demographical differences in MSM communities across different Chinese geographical regions. This is consistent with the fact that Southwest China has higher HIV prevalence than its syphilis prevalence, which is distinctively different to the rest of the country. Prevalence estimates of these two epidemics were significantly increasing during 2003 to 2008 and this also contributes to the heterogeneity. We further found that articles published in English language were more likely to report higher syphilis prevalence estimates than Chinese articles. However, sampling size, study base and sampling method cannot explain the variation in the results. Due to small numbers of studies available for HIV-syphilis co-infection, we were not able to identify any study factors that were associated with the variation in HIV-syphilis co-infection prevalence estimates.

Our estimates of the latest national HIV prevalence (4.7%) and current syphilis prevalence (13.5%) are higher than a recent estimate of 2.5% and 9.1% [95]. This previous meta-analysis study did not capture the chronological trend of the epidemics by pooling all the HIV and syphilis prevalence estimates from 2001 to 2008 together and therefore likely underestimated current prevalence. Gao et al estimated the syphilis prevalence among Chinese MSM to be 9.1% [95]; however, their selection criteria did not exclude studies with single syphilis test and hence their estimate is likely to be a mixed prevalence of current syphilis infection and people ever infected with syphilis. On contrast, our study employs a distinguish approach to ensure the analysis of the actual syphilis-infected prevalence. In addition, our study contains more than 70 collated studies from different regions during 2003–2008, the number of prevalence estimates included in quantitative synthesis was tripled (95 and 70 for HIV and syphilis prevalence estimates) in comparison with the study from Gao et al (31 and 24 for HIV and syphilis prevalence estimates) [95]. Hence, our study provides a more thorough estimate of the level and trend of HIV epidemics among MSM across China. In comparison with the Gao et al study, our study provides newer information on: (1) chronologically increasing trend of HIV and syphilis prevalence; (2) trend and current prevalence of HIV-syphilis co-infection (3) the correlation of HIV and syphilis epidemic among MSM in China. To our knowledge, this is the first time that such newer information is provided through a quantitative and qualitative synthesis.

Our findings are also consistent with other epidemiological studies. When stratified by year periods, our national HIV prevalence estimates (1.3% in 2003–2004, 2.4% in 2005–2006 and 4.7% in 2007–2008) are similar to earlier estimates by Tang et al. (0.5–1.5% in 2003–2004 and 2.8–3.0% in 2005–2006 [96]) and a large HIV survey conducted in eighteen Chinese cities (2.3% in 2006 and 5.0% in 2008) [97]. In contrast, Lin et al. estimated a 4.5% annual increase in syphilis prevalence [98], which is much higher than our estimate (1.2%). As Lin's analysis included only four studies for the investigated period, this result may be biased. Our study, with 70 syphilis prevalence estimates distributed between 2003 and 2008 provides a more thorough estimate of the national level and growth rate of syphilis infection among Chinese MSM.

With substantial increases in HIV and syphilis infection, we also observed an increase in prevalent HIV-syphilis co-infections (1.4% during 2005–2006 to 2.7% during 2007–2008). The co-infection percentage demonstrates the interaction of the two epidemics among Chinese MSM. As the presence of syphilis facilitates greater risk of HIV transmission, the increasing trend of co-infections may imply a larger MSM sub-population with a greater HIV infectivity and/or susceptibility. It is possible that syphilis infections are driving some of the increase in the HIV epidemic among MSM in China. Greater syphilis prevalence than HIV prevalence found in most Chinese regions is consistent with studies from other settings [43], [63], [99], [100] but the reverse result was observed in Southwest China. We postulate two possible reasons for this observation. First, southwest China is traditionally a region with high injecting drug use. It is estimated that over 50% of China's people who inject drugs (PWID) are concentrated in the Yunnan and Sichuan province in Southwest China [101]. High HIV prevalence among injecting drug users (IDU) were recorded in Southwest China, in particular, it was 28.4% in Yunnan province and 8.8% in the neighbouring Sichuan province in 2007 [102], [103]. These are substantially higher than the national average of 7.0% [104]. It is also documented that about 8.3% Chinese MSM and 44% male sex workers [105] have drug use history and the percentage is generally expected to be higher in Southwest China, where drug usage is more prevalent [106], [107]. Although any direct evidences on injecting behaviours among MSM in Southwest China remains absent, a number of studies have indicated that injection sharing among MSM could be a potential bridge of HIV transmission from IDU to MSM in this region [107], [108]. It is therefore likely that HIV among MSM in this region is not only driven by homosexual exposure but also by their injecting behaviours. Second, the first large HIV outbreaks in China were observed among PWID in the Southwest province of Yunnan in the late 1980s; the higher HIV prevalence in Southwest China could simply be due to the much earlier onset of the epidemic in this region.

The increases in prevalence of HIV and syphilis among MSM in China indicate that the twin epidemics are likely affecting similar population in China. Studies showed that both epidemics have a rapid growing rate than any other vulnerable populations in China [16], [109]. It is important to establish public health responses that are likely to be effective, acceptable and feasible to implement. HIV-syphilis co-infections are significant in that they may act as biological co-factors to increase their respective transmission rates [110], [111]. In addition, due to similar modes of transmission, single campaigns can be designed with the objective of reducing the spread of both syphilis and HIV. China has experience developing strategies targeting co-infections. In recent years, China's HIV/AIDS programs have been funded by various international sponsors [112]. There have been HIV-syphilis integration programs (such as the Plum Blossom Project) supported by the National Institutes of Health Office and the American Recovery and Reinvestment Act to provide free HIV and syphilis testing in seven public clinics in Guangdong province. However, these programs are not MSM-specific. This framework could be a starting point for developing a strategy and action plan for effectively reducing the incidence and burden of HIV, syphilis and other sexual and general health issues for MSM in China.

## Supporting Information

Table S1 **PRISMA checklist.**
(DOC)

Table S2 **Studies reporting both HIV and syphilis prevalence among men who have sex with men in China.** Each section represents different Chinese regions. The lines in each section separate different time periods: 2003–2004, 2005–2006 and 2007–2008.
(DOCX)
