# Supplementary material for: HIV and Syphilis Co-Infection Increasing among Men Who Have Sex with Men in China: A Systematic Review and Meta-Analysis
Source: PLoS One. 2011 Aug 15;6(8):e22768. doi: 10.1371/journal.pone.0022768 (PMC3156129; doi:10.1371/journal.pone.0022768)
Supplement: Table S2 — Studies reporting both HIV and syphilis prevalence among men who have sex with men in China. Each section represents different Chinese regions. The lines in each section separate different time periods: 2003–2004, 2005–2006 and 2007–2008. (DOCX) [file pone.0022768.s002.docx]

**Table S2: Studies reporting both HIV and syphilis prevalence among men who have sex with men in China.** Each section represents different Chinese regions. The lines in each section separate different time periods: 2003-2004, 2005-2006 and 2007-2008.

| **First author,**  **published year ^** | **Study design** | | | | | | | **Prevalence %(n)** | | |
| --- | --- | --- | --- | --- | --- | --- | --- | --- | --- | --- |
|  | **Study Period** | **Study**  **Location** | **Age range (Mean)** | **Study base** | **Sampling method ^&^** | **Sample size** | **Syphilis testing method ^¶^** | **Syphilis infection** | **HIV**  **infection** | **HIV-syphilis**  **Co-infection** |
| ***East China*** |  |  |  |  |  |  |  |  |  |  |
| Jiang J, 2006 [1] | 2003 | 5 cities in Jiangsu (Nanjing, Yangzhou, Changzhou, Wuxi, Suzhou) | 18-29 | MSM venues | VBS | 144 | RPR, TPPA | 6.94% (10) * | 0.00% (0) | - |
| Jiang SM, 2006 [2] | 2004 | Weihai | 19-59 (30) | - | - | 19 | Unspecified | 15.79% (3) | 0.00% (0) | - |
| Cai GF, 2008 [3] | 2006 | Zhejiang | 16-46 | MSM venues | - | 59 | ELISA, TRUST, TPPA | 8.47% (5) * | 3.39% (2) | - |
| Cai X, 2007 [4] | 2006 | Liaocheng | 18-38 (26) | Participates in a health symposium | - | 94 | ELISA, TPPA | 6.38% (6) * | 1.06% (1) | - |
| Choi KH, 2007 [5] | 2004-2005 | Shanghai | 18-56 (28) | MSM venues | Snowball | 475 | TRUST, TPPA | 13.47% (64) * | 1.47% (7) | 0.42% (2) † |
| Fu LJ, 2007 [6] | 2006 | Zhejiang | 19-41 (26) | Peer referral | Snowball | 51 | Unspecified | 11.76% (6) | 0.00% (0) | - |
| Guo H, 2009 [7] | 2006 | Nanjing | N/A | MSM venues | - | 296 | TPPA, RPR | 26.69% (79) * | 4.73% (14) | 2.70% (8) † |
| Liao MZ, 2006 [8] | 2005 | 3 cities in Shandong (Yantai, Jinan, Weihai) | 18-73 (26) | MSM venues | - | 142 | Unspecified | 4.92% (7) | 4.92% (7) | - |
|  |  |  |  |  |  |  |  |  |  |  |
| Wu J, 2008 [9] | 2006 | Shanghai | 18-54 (27) | MSM venues | - | 203 | RPR, TPPA | 10.34% (21) * | 4.43% (9) | 2.96% (6) † |
| Xu J, 2007 [10] | 2005 | Hefei | 18-42 (23) | Peer referral, internet advertisement | RDS | 223 | TRUST, TPPA | 8.07% (18) * | 1.79% (4) | - |
| Xu J, 2007 [11] | 2005 | Hefei | 15-24 (21) | Peer referral, internet advertisement | RDS | 199 | RPR, TPPA | 13.07% (26) * | 1.01% (2) | - |
| Zhu JL, 2007 [12] | 2005 | Hefei | 18-29 (20) | Peer referral, internet advertisement | RDS | 121 | TRUST, TPPA | 13.22% (16) * | 1.65% (2) | - |
| Zhu YW, 2007 [13] | 2006 | Jinan | 17-66 (25) | CDC Clinic | - | 282 | TPPA | 9.57% (27) | 1.06% (3) | - |
| Bao YG, 2009 [14] | 2008 | Hangzhou | N/A | - | RDS | 474 | RPR, TPPA | 16.03% (76) * | 5.06% (24) | - |
| Bao YG, 2009 [14] | 2008 | Shanghai | N/A | - | RDS | 465 | RPR, TPPA | 12.26% (57) * | 7.53% (35) | - |
| Bao YG, 2009 [14] | 2008 | Nanjing | N/A | - | RDS | 430 | RPR, TPPA | 11.86% (51) * | 4.65% (20) | - |
| Bao YG, 2009 [14] | 2008 | Qingdao | N/A | - | RDS | 350 | RPR, TPPA | 4.00% (14) * | 1.14% (4) | - |
| Feng JF, 2008 [15] | 2007 | Zhejiang | 18-48 (28) | MSM venues | - | 73, 77 | ELISA | 24.66% (18) | 3.90% (3) | 0.00% (0) |
| Guo H, 2009 [7] | 2007 | Yangzhou | N/A | MSM venues | - | 137 | TPPA, RPR | 29.93% (41) * | 8.03% (11) | 5.84% (8) † |
| Han XY, 2007 [16] | 2005-2007 | Jinan | 17-62 | CDC Clinic | - | 635 | TPPA | 9.76% (62) | 2.20% (14) | - |
| Hu J, 2008 [17] | 2007 | Huai'an | 19-54 | Internet recruitment | - | 65 | TRUST, RPR, TPPA | 6.15% (4) * | 1.54% (1) | - |
| Lu ZX, 2009 [18] | 2007 | Yancheng | 17-53 | Internet recruitment | - | 61 | Unspecified | 1.64% (1) | 1.64% (1) | - |
| Lu ZX, 2009 [18] | 2008 | Yancheng | 17-53 | Internet recruitment | - | 68 | Unspecified | 7.35% (5) | 4.41% (3) | - |
| Ruan S, 2008 [19] | 2007 | Jinan | N/A | Peer referral | RDS | 428 | Unspecified | 4.91% (21) | 0.70% (3) | - |
| Yang H, 2010 [20] ^ | 2008 | Nanjing | N/A | MSM venues | RDS | 397/416 | RPR, TPPA | 12.34% (49)* | 4.57% (19) | - |
| ***Northeast China*** |  |  |  |  |  |  |  |  |  |  |
| Gu Y, 2004 [21] | 2003 | Shenyang | 16-49 | MSM venues | - | 202 | TPPA | 20.79% (42) | 0.99% (2) | - |
| Wang J, 2004 [22] | 2003 | Dalian | 17-40 | MSM venues, internet recruitment | - | 157 | TRUST, RPR, TPPA | 1.27% (2) * | 0.00% (0) | - |
| Wang J, 2008 [23] | 2006 | Harbin | N/A | MSM venues | Convenience | 401 | RPR | 8.98% (36) | 1.00% (4) | - |
| Xiao Y, 2010 [24] | 2006 | Liaoning | 15-68 | MSM venues, internet advertisement, community outreach, peer referral | VBS, snowball | 1243 | RPR, TPPA | 8.37% (104) * | 1.93% (24) | - |
| Xiao Y, 2010 [24] | 2006 | Heilongjiang | 15-68 | MSM venues, internet advertisement, community outreach, peer referral | VBS, snowball | 809 | RPR, TPPA | 9.77% (79) * | 0.74% (6) | - |
| Xiao Y, 2010 [24] ^ | 2006 | Jilin | 15-68 | MSM venues, internet advertisement, community outreach, peer referral | VBS, snowball | 1211 | RPR, TPPA | 12.80% (155) * | 0.83% (10) | - |
| Xu JJ, 2010 [25] ^ | 2006 | Shenyang | 18-60 | Health education outreach (NGO) | - | 218/231 | RPR, TPPA | 33.49% (73) * | 5.63% (13) | - |
| Bao YG, 2009 [14] | 2008 | Shenyang | N/A | - | RDS | 450 | RPR, TPPA | 11.78% (53) * | 8.67% (39) | - |
| Bao YG, 2009 [14] | 2008 | Harbin | N/A | - | RDS | 451 | RPR, TPPA | 19.07% (86) * | 4.43% (20) | - |
| Sun M, 2009 [26] | 2008 | Dalian | 18-59 | MSM venues, VCT sites | - | 401 | RPR, ELISA | 17.46% (70) * | 4.24% (17) | - |
| Xu H, 2009 [27] | 2008 | Anshan | N/A | MSM venues | Snowball | 400 | TPPA | 8.75% (35) | 2.75% (11) | - |
| Xu ZH, 2009 [28] | 2007 | Tieling | 16-45 | MSM venues | - | 34 | Unspecified | 2.94% (1) | 0.00% (0) | - |
| Zhang JD, 2008 [29] | 2007 | Harbin | 17-59 (33) | MSM venues | - | 121 | RPR, TPPA | 15.70% (19) * | 3.31% (4) | - |
| Zhao HP, 2009 [30] | 2008 | Harbin | 17-52 (25) | VCT Clinic | - | 89 | ELISA , WB | 15.73% (14) * | 2.25% (2) | - |
| ***North China*** |  |  |  |  |  |  |  |  |  |  |
| Ma X, 2007 [31] | 2004 | Beijing | ≥18 | Peer referral | RDS | 325 | ELISA, RPR | 7.69% (25) * | 1.54% (5) | - |
| Liu H, 2007 [32] | 2005 | Beijing | 18-55 (27) | MSM venues | - | 416 | RPR, TPPA | 10.34% (43) * | 1.68% (7) | 0.48% (2) † |
| Lu HY, 2008 [33] | 2006 | Beijing | 18-44 (26) | CDC Clinic | Snowball | 200 | RPR, ELISA, TPPA | 24.00% (48) * | 6.50% (13) | - |
| Ma J, 2007 [34] | 2006 | Tianjin | N/A (25) | Internet recruitment | - | 62 | Unspecified | 16.13% (10) | 4.84% (3) | - |
| Ma X, 2007 [31] | 2005 | Beijing | ≥16 | Peer referral | RDS | 427 | ELISA, RPR | 12.65% (54) * | 6.09% (26) | - |
| Ma X, 2007[31] | 2006 | Beijing | ≥16 | Peer referral | RDS | 540 | ELISA, RPR | 10.00% (54) * | 6.85% (37) | - |
| Ruan Y, 2007 [35] | 2005 | Beijing | 17-54 (26) | MSM venues, internet advertisement, peer referral | - | 526 | ELISA, TPPA | 11.22% (59) * | 3.23% (17) | - |
| Wang CH, 2007 [36] | 2006 | Chengde | 15-28 (22) | MSM venues, internet advertisement | Snowball | 82 | ELISA | 21.95% (18) | 3.66% (3) | - |
| Xiao Y, 2010 [24] | 2006 | Inner Mongolia | 15-68 | MSM venues, internet advertisement, community outreach, peer referral | VBS, snowball | 167 | RPR, TPPA | 29.34% (49) * | 0.00% (0) | - |
| Zhang X, 2007 [37] | 2006 | Beijing | 18-55 (26) | VCT Clinic | - | 735 | RPR, TPHA | 7.21% (53) * | 2.18% (16) | - |
| Bao YG, 2009 [14] | 2008 | Beijing | N/A | - | RDS | 607 | RPR, TPPA | 18.62% (113) * | 5.93% (36) | - |
| Bao YG, 2009 [14] | 2008 | Tianjin | N/A | - | RDS | 419 | RPR, TPPA | 18.85% (79) * | 6.44% (27) | - |
| Guo Y, 2009 [38] | 2007 | Tianjin | 17-57 (28) | MSM venues | - | 204 | RPR, TPPA | 18.63% (38) * | 5.88% (12) | 3.43% (7) † |
| Liang L, 2009 [39] | 2008 | Hebei | N/A | VCT Clinic, peer referral | Snowball | 450 | TRUST, TPPA | 7.56% (34) * | 2.22% (10) | 0.67% (3) † |
| Qu L, 2009 [40] | 2008 | Inner Mongolia | 18-63 (27) | MSM venues | - | 604 | RPR, TPPA | 20.03% (121) * | 3.31% (20) | - |
| Ruan Y, 2009 [41] | 2006-2007 | Beijing | 18-62 | Peer referral, community outreach, internet | - | 541 | EIA, TPPA | 19.80% (107) * | 4.80% (26) | - |
| Wang XL, 2009 [42] | 2008 | Tangshan | 19-53 | - | - | 77 | ELISA, RPR | 29.87% (23) * | 7.79% (6) | - |
| ***South Central China*** |  |  |  |  |  |  |  |  |  |  |
| Cai WD, 2005 [43] | 2004 | Shenzhen | 17-58 (26) | MSM venues | - | 261 | TPPA | 18.01% (47) | 1.53% (4) | - |
| He Q, 2005 [44] | 2003 | Guangzhou | N/A | Peer referral, internet advertisement | Snowball | 117 | ELISA, RPR | 11.11% (13) * | 1.71% (2) | - |
| He Q, 2008 [45] | 2004 | Guangzhou | 16-66 (30) | Newspaper advertisement, the Internet, referral by acquaintance, television advertisement | Convenience | 201 | Unspecified | 1.99% (4) | 0.00% (0) | - |
| Tao XY, 2004 [46] | 2003 | Shenzhen | 17-47 (24) | MSM venues | - | 114 | Unspecified | 2.63% (3) | 1.75% (2) | - |
| Cai YM, 2008 [47] | 2005 | Shenzhen | 18-50 (28) | MSM venues | - | 242 | TRUST, TPPA | 9.92% (24) * | 1.65% (4) | 1.24% (3) † |
| Chen SH, 2007 [48] | 2006 | Nanning | 18-45 (26) | Website | Convenience | 185 | TRUST, TPPA | 10.81%(20) * | 0.00% (0) | - |
| He Q, 2008 [45] | 2006 | Guangzhou | ≥18 | Newspaper advertisement, the Internet, referral by acquaintance, television advertisement | Convenience | 423 | Unspecified | 4.90% (21) | 1.70% (7) | - |
| Bao YG, 2009 [14] | 2008 | Guangzhou | N/A | - | RDS | 379 | RPR, TPPA | 17.41% (66) * | 5.28% (20) | - |
| Bao YG, 2009 [14] | 2008 | Wuhan | N/A | - | RDS | 456 | RPR, TPPA | 21.49% (98) * | 5.04% (23) | - |
| Bao YG, 2009 [14] | 2008 | Haikou | N/A | - | RDS | 98 | RPR, TPPA | 12.24% (12) * | 2.04% (2) | - |
| Feng F, 2009 [49] | 2008 | Haikou | 18-55 (28) | MSM venues, internet recuriment | Snowball, convenience | 104 | RPR, TRUST, TPPA | 12.50% (13) * | 1.92% (2) | - |
| Feng TJ, 2008 [50] | 2005-2007 | Shenzhen | 17-68 (29) | MSM venues & clinic | - | 1376 | TRUST, TPPA | 19.19% (264) * | 3.34% (46) | - |
| Hong FC, 2009 [51] | 2007 | Shenzhen | N/A | MSM venues, STD clinic | - | 1146 | TRUST, TPPA | 20.70% (241) * | 3.66% (42) | - |
| Lan GH, 2009 [52] | 2008 | Guangxi | 18-59 (30) | - | - | 179 | RPR, TRUST | 7.82% (14) * | 1.68% (3) | - |
| Shi WD, 2009 [53] | 2008 | Wuhan | 18-60 (28) | Peer referral | RDS | 456 | RPR, TPPA | 21.49% (98) * | 5.04% (23) | - |
| Wen F, 2010 [54] | 2008 | Guangzhou | 18-64 (28) | MSM venues, internet | Snowball | 452 | RPR, TPPA | 5.97% (27) * | 4.87% (22) | 1.33% (6) † |
| Weng YQ, 2009 [55] | 2008 | Guangxi | 18-57 (28) | MSM venues, VCT Clinic | Snowball | 239 | RPR | 4.60% (11) | 3.35% (8) | - |
| Zhong F, 2009 [56] | 2008 | Guangzhou | 18-51 | VCT Clinic | RDS | 379 | RPR, TPPA | 17.94% (68) * | 5.28% (20) | - |
| ***Northwest China*** |  |  |  |  |  |  |  |  |  |  |
| Li Y, 2007 [57] | 2006 | Lanzhou | 18-61 (30) | MSM venues | Snowball | 264 | RPR, TPPA | 2.65% (7) * | 0.76% (2) | - |
| Xiao Y, 2010 [24] | 2006 | Gansu | 15-68 | MSM venues, internet advertisement, community outreach, peer referral | VBS, snowball | 477 | RPR, TPPA | 1.89% (9) * | 0.42% (2) | - |
| Xiao Y, 2010 [24] | 2006 | Ningxia | 15-68 | MSM venues, internet advertisement, community outreach, peer referral | VBS, snowball | 76 | RPR, TPPA | 1.32% (1) * | 0.00% (0) | - |
| Yang SP, 2008 [58] | 2006 | Hami Prefecture | 15-57 (28) | - | - | 61 | RPR | 1.64% (1) | 3.28% (2) | - |
| Bao YG, 2009 [14] | 2008 | Xi’an | N/A | - | RDS | 98 | RPR, TPPA | 17.80% (79) * | 3.29% (15) | - |
| Miao ZF, 2009 [59] | 2008 | Yinchuan | 18-55 (28) | MSM venues, , Internet | Snowball | 312 | Unspecified | 2.88% (9) | 1.92% (6) | - |
| Qiao XW, 2009[60] | 2008 | Lanzhou | 16-68 | VCT sites | - | 619 | TRUST, TPPA | 10.34% (64) * | 4.85% (30) | 1.94% (12) † |
| Zhang M, 2009 [61] | 2008 | Ürümqi | 18-54 (27) | - | RDS | 231 | RPR, TPPA | 10.82% (25) * | 2.16% (5) | - |
| Zhang Y, 2008 [62] | 2007 | Ürümqi | N/A | Internet recruitment | - | 143 | RPR, TPPA | 9.79% (14) * | 5.59% (8) | - |
| ***Southwest China*** |  |  |  |  |  |  |  |  |  |  |
| Lu CG, 2006 [63] | 2003-2005 | Guizhou | 17-81 | Peer referral | Snowball | 200 | Unspecified | 1.00 (2) | 2.00 (4) | - |
| Lu CG, 2006 [64] | 2003-2005 | Guiyang | 15-81 | MSM venues | Snowball | 276 | Unspecified | 0.72 (2) | 2.17 (6) | - |
| Feng L, 2009 [65] | 2006 | Chongqing | N/A | MSM venues, peer referral, web-based recruitment | VBS, ABCS | 1000 | RPR, TPPA | 9.30 (93) * | 10.40 (104) | 1.70 (17) † |
| Zhou J, 2008 [66] | 2006 | Guiyang | 15-49 (24) | Peer referral, MSM hotlines, internet recruitment | - | 406 | RPR, TPPA | 1.97 (8) * | 5.91 (24) | 0.73 (3) † |
| Bao YG, 2009 [14] | 2008 | Chongqing | N/A | - | RDS | 617 | RPR, TPPA | 7.29% (45) * | 10.86% (67) | - |
| Bao YG, 2009 [14] | 2008 | Kunming | N/A | - | RDS | 450 | RPR, TPPA | 7.33% (33) * | 11.78% (53) | - |
| Ding XB, 2010 [67] | 2008 | Chongqing | 18-67 (26) | CDC clinic | Snowball | 743 | RPR, TPPA | 8.48% (63) * | 16.55% (123) | 4.31% (32) † |
| Feng L, 2009 [65] | 2007 | Chongqing | N/A | MSM venues, peer referral, web-based recruitment | VBS, ABCS | 1044 | RPR, TPPA | 8.52% (89) * | 12.55% (131) | 2.68% (28) † |
| Feng Y, 2010 [68] | 2007 | Chengdu | 16-44 | MSM venues | Snowball | 513 | ELISA | 28.07 (14) | 9.55% (49) | - |
| Wang Y, 2008 [69] | 2006-2007 | Mianyang | 16-57 (25) | - | RDS | 81 | RPR, TPPA | 8.60% (7) * | 0.00% (0) | - |
| Wang Y, 2009 [70] | 2007 | Mianyang | 16-40 | MSM venues | RDS | 111 | RPR, TPPA | 17.12% (19) * | 7.21% (8) | 3.60% (4) † |
| Xiao, Y, 2009 [71] | 2007 | Chongqing | 18-68(28) | MSM venues, internet advertisement, community outreach, peer referral | VBS, snowball | 1692 | RPR, TPPA | 8.57 (145) * | 10.76% (182) | - |

^^^  represents cohort study.

^&^ RDS: Respondent Driven Sampling; VBS: Venue-based sampling; ABCS: area-based conveience sampling

* Have both screening and confirmatory tests for syphilis and hence used in meta-analyses to estimate the prevalence of syphilis among MSM during 2003-2008.

† Used in meta-analyses to estimate the prevalence of HIV-syphilis co-infection among MSM during 2003-2008.

**^¶^** EIA: Enzyme immunoassay; ELISA: Enzyme-linked immunosorbent assay; RPR: Rapid Plasma Reagin; TPPA: Treponema pallidum Particle Agglutination assay; TPHA: Treponema pallidum hemagglutination assays; TRUST: Toluidine red unheated serum test; Unspecified: method of syphilis testing did not mention in the study.

**References**

1. Jiang J, Cao N, Zhang J, Xia Q, Gong X, et al. (2006) High prevalence of sexually transmitted diseases among men who have sex with men in Jiangsu Province, China. Sex Transm Dis 33: 118-123.

2. Jiang SM, Li JZ, Li RY, Zhang HJ, Xiao HC (2006) [Investigation on unprotected sex and AIDS knowledge among men who have sex with men in Weihai city]. Prev Med Trib 12: 763-764.

3. Cai GF, Ma QQ, Pan XH, Fu LJ, Xu WX, et al. (2008) [HIV/AIDS Related Knowledge, Attitude, Practice and HIV/STD Infection among MSM in Two Cities of Zhejiang Province]. China Preventive Medicine 9: 482-485.

4. Cai X (2007) [The Analysis of Risk Behaviors of MSM in Liaocheng and the Serological Detection for Anti-HIV, Anti-TP, and Anti-HCV Antibodies in 2006]. Preventive Medicine Tribune 13: 888-890.

5. Choi KH, Ning Z, Gregorich SE, Pan QC (2007) The influence of social and sexual networks in the spread of HIV and syphilis among men who have sex with men in Shanghai, China. J Acquir Immune Defic Syndr 45: 77-84.

6. Fu LJ, Fang YR, Guo TY (2007) [Investigation of the sexual behaviors among the MSM in Shaoxing City of Zhejiang Province]. Disease Surveillance 22: 818-819.

7. Guo H, Wei JF, Yang H, Huan X, Tsui SK, et al. (2009) Rapidly increasing prevalence of HIV and syphilis and HIV-1 subtype characterization among men who have sex with men in Jiangsu, China. Sex Transm Dis 36: 120-125.

8. Liao MZ, Liu XZ, Fu JH, Qian YS, Zhang XF (2006) [Analysis of data of behavioral surveillance in men who have sex with men(MSM) in Shandong Province]. Chinese Journal of AIDS & STD 12: 530-532.

9. Wu J, Chen L, Fan HL, Ruan Y (2008) [A survey on the prevalence of HIV-1 and syphilis infection and characteristics of sexual behaviors in MSM (men who have sex with men) living in Shanghai]. J Diagn Concepts Pract 7: 296-299.

10. Xu J, Wu ZJ, Zhang HB, Zhu JL, Wu HH, et al. (2007) [Study On The Status Of HIV Infection And Syphilis Infection Among MSM In Hefei And Its Correlation With The Social And Sexual Partner Characteristics Of MSM]. Modern Preventive Medicine 34: 3009-3011, 3014.

11. Xu J, Zhang HB, Zheng YJ, Wang J, Zhu YB, et al. (2007) [The prevalence of syphilis and HIV infection among young men who have sex with men in Hefei city]. Chinese Journal of Behavioral Medical Science 16: 205-207.

12. Zhu JL, Zhang HB, Wu HH (2007) [High risk sexual behavior and HIV/STD infection rate among 122 MSM from students]. Chin J AIDS STD 13: 350-352.

13. Zhu YW, Ruan SM, Yang H, Wang MH, Zhang CQ, et al. (2007) [Investigation on AIDS Related Knowledge Risk Behavior and HIV Infection in MSM in Ji'nan City]. Preventive Medicine Tribune 13: 490-492.

14. Bao YG, Zhang YH, Zhao JK, Sun JP, Tan HZ (2009) [HIV infection and KAP status among men who have sex with men in 14 Chinese cities]. Zhonghua Yu Fang Yi Xue Za Zhi 43: 981-983.

15. Feng JF, Lin HJ, Zhang YF, Qiu DH, Wu QH, et al. (2008) [Investigation on the related knowledge, behavior and infection of HIV/Syphilis among men who have sex with men in Taizhou city]. Shanghai Journal of Preventive Medicine 20: 531-533, 541.

16. Han XY, Liu LZ, Zhang W, Ma DN, Chu Q, et al. (2007) [Investigation on the Related Nosogenic Infection among 635 Men Who Have Sex with Men in Ji'nan]. Preventive Medicine Tribune 13: 1091-1092.

17. Hu J, Wu L, Zhang Z, Fan W (2008) [Investigation of STD/AIDS infection, related knowledge and high-risk sexual behavior among MSM in Huaian city]. Journal of Public Health and Preventive Medicine 19: 44 - 45.

18. Lu ZX, Ge N, Gao HR, Yan LL, Yu WX (2009) [Investigation on AIDS Related Knowledge, Behavior and Infection among Men who Have Sex with Men (MSM) in Yancgeng City from 2007 to 2008]. Prev Med Trib 15: 702-703.

19. Ruan S, Yang H, Zhu Y, Ma Y, Li J, et al. (2008) HIV prevalence and correlates of unprotected anal intercourse among men who have sex with men, Jinan, China. AIDS Behav 12: 469-475.

20. Yang H, Hao C, Huan X, Yan H, Guan W, et al. (2010) HIV Incidence and Associated Factors in a Cohort of Men Who Have Sex With Men in Nanjing, China. Sex Transm Dis 37: 208-213.

21. Gu Y, Qu P, Xu L, Luo M, Wang X, et al. (2004) [Survey of knowledge, attitude, behavior and practice related to STI/HIV among male homosexuality in Shenyang]. Chin J Public Health 20: 573-574.

22. Wang J, Dong W, Li R, Zhao Z, Xiong B, et al. (2004) [A survey of HIV/STD infection and behaviors among MSM]. Chinese Journal of Public Health 20: 1377 - 1378.

23. Wang J, Luo C, Wen YC (2008) [HIV infection and related risk behaviors among MSM in Harbin]. Chinese Journal of AIDS & STD 14: 75.

24. Xiao Y, Sun J, Li C, Lu F, Allen KL, et al. (2010) Prevalence and correlates of HIV and syphilis infections among men who have sex with men in seven provinces in China with historically low HIV prevalence. J Acquir Immune Defic Syndr 53 Suppl 1: S66-73.

25. Xu JJ, Zhang M, Brown K, Reilly K, Wang H, et al. (2010) Syphilis and HIV Seroconversion Among a 12-Month Prospective Cohort of Men Who Have Sex With Men in Shenyang, China. Sex Transm Dis.

26. Sun ML, Li DJ, Jin W, Jiang J, Guan L (2009) [Investigation on the Infection of HIV, HCV, Syphilis and HBV among MSM in Dalian City in 2008]. Preventive Medicine Tribune 15: 1074 - 1075.

27. Xu H, Hu W, Zhao HY (2009) [AIDS survey of MSM homosexuality population in Anshan]. Chinese Journal of Health Laboratory Technology 19: 658,713.

28. Xu ZH (2009) [Investigation on AIDS knowledge and behaviors among men who have sex with men in the city of Tieling]. Chin J Public Health 25: 226.

29. Zhang JD, Sun YB, Wang S, Liu HJ, Li LJ, et al. (2008) [HIV and syphilis infections among 121 men who have sex with men in Harbin]. Chinese Journal of AIDS & STD 14: 69.

30. Zhao HP, Wang GM, Liu HY (2009) [The Investigation Report of Male-to-male Contact During the Voluntary Consulation in Daolin District of Harebin]. Guide of China Medicine 7: 181-183.

31. Ma X, Zhang Q, He X, Sun W, Yue H, et al. (2007) Trends in Prevalence of HIV, Syphilis, Hepatitis C, Hepatitis B, and Sexual Risk Behavior Among Men Who Have Sex With Men. Results of 3 consecutive respondent-driven sampling surveys in Beijing, 2004 through 2006. J Acquir Immune Defic Syndr 45: 581-587.

32. Liu H, Wang N, Zhang Q, Shao Y, Wu T, et al. (2007) [Study of HIV and syphilis infection situation and sexual behavioral characteristics among 416 MSM]. Chinese Journal of AIDS & STD 13: 230-232, 234.

33. Lu HY, Ma XY, Liu YC, Zhang QY, Hei FX, et al. (2008) [A survey of HIV/STDs prevalence in 200 MSM and related factors in Beijing]. Chinese Journal of AIDS & STD 14: 467-470.

34. Ma J, Guo J (2007) [Internet survey of high risk sexual behaviors and sexually transmitted diseases among male homosexuals in Tianjin]. Modern Preventive Medicine 34: 3928-3931.

35. Ruan Y, Li D, Li X, Qian HZ, Shi W, et al. (2007) Relationship between syphilis and HIV infections among men who have sex with men in Beijing, China. Sex Transm Dis 34: 592-597.

36. Wang CH, Jiang MJ, Lu GJ, Bin G (2007) [HIV epidemic spread between male homosexuals in the city of Chengde]. Chinese Journal of Health Laboratory Technology 17: 883-884.

37. Zhang X, Wang C, Hengwei W, Li X, Li D, et al. (2007) Risk factors of HIV infection and prevalence of co-infections among men who have sex with men in Beijing, China. AIDS 21 Suppl 8: S53-57.

38. Guo Y, Zhu XK, Xia JH, Dong XY (2009) [Study on HIV/syphilis infections among men who have sex with men and their behavioral feature]. Chinese Journal of AIDS & STD 15: 50-51, 71.

39. Liang L, Chen ZQ, Miao XF, Li Bj, Bai GY, et al. (2009) [An investigation of HIV infections among men who have sex with men]. Hebei Medical Journal 31: 1991-1992.

40. Qu L, Yang JY, Zhang XG, Yang YR, Bao ZQ (2009) [Study on HIV and syphilis infections among men who have sex with men in selected cities of Inner Mongolia]. Chin J Epidemiol 30.

41. Ruan Y, Luo F, Jia Y, Li X, Li Q, et al. (2009) Risk factors for syphilis and prevalence of HIV, hepatitis B and C among men who have sex with men in Beijing, China: implications for HIV prevention. AIDS Behav 13: 663-670.

42. Wang XL, He JK, Su HH, Cao HZ, Zhang QJ, et al. (2009) [HIV and syphilis infection in male homosexuality in Tangshan city]. Chinese Journal of Public Health 25: 787-788.

43. Cai WD, Fen TJ, Tan JQ, Chen L, Shi XD, et al. (2005) [A Survery Of The Characteristics And STD/HIV Infection Of Homosexuality In Shenzhen]. Modern Preventive Medicine 32: 328-330.

44. He Q, Wang Y, Lin P, Zhang Z-b, Zhao X-x, et al. (2005) [KAP study on AIDS among men who have sex with men in Guangzhou, Guangdong province]. Chinese Journal of Disease Control & Prevention 9: 106-108.

45. He Q, Wang Y, Li Y, Zhang Y, Lin P, et al. (2008) Accessing men who have sex with men through long-chain referral recruitment, Guangzhou, China. AIDS Behav 12: S93-96.

46. Tao XY, Cai WD, Cai YM, Wei AY, Huang GW, et al. (2004) [Survey of related high risk sexual behaviors of MSM in Shenzhen city]. Modern Preventive Medicine 31: 247-248.

47. Cai YM, Liu H, Pan P, Hong FC, Zhou H (2008) [Epidemiological Investigation on the HIV/Syphilis among men who have sex with men from gay saunas and bars in Shenzhen]. Modern Preventive Medicine 35: 2203-2205.

48. Chen S, Zhou J, Zhu JJ (2007) [Investigation of STI among some Men Who Have Sex with Men in Nanning City in 2006]. Prev Med Trib 13: 772-774.

49. Feng F, Wang ZQ, Huang SP, Lu JG, Lin ZW, et al. (2009) [Investigation on aids knowledge, attitude and practice characteristics of MSM group and HIV/syphilis infection situation]. Modern Preventive Medicine 36: 2902-2903, 2909.

50. Feng TJ, Liu XL, Cai YM, Pan P, Hong FC, et al. (2008) Prevalence of syphilis and human immunodeficiency virus infections among men who have sex with men in Shenzhen, China: 2005 to 2007. Sex Transm Dis 35: 1022-1024.

51. Hong FC, Zhou H, Cai YM, Pan P, Feng TJ, et al. (2009) Prevalence of syphilis and HIV infections among men who have sex with men from different settings in Shenzhen, China: implications for HIV/STD surveillance. Sex Transm Infect 85: 42-44.

52. Lan GH, Liu W, Zhu QY, Liang FX, Zhou Y (2009) [To analyze the surveillance results of immigrant MSM in Guangxi]. Applied Prev Med 15: 168-170.

53. Shi WD, Li G, Yang T, Zhou W, Liu PL, et al. (2009) [Survey of High Risk Sexual Behaviors and HIV,Syphilis,HCV among 456 Male Homosexuals in Wuhan]. Medicine and Society 22: 42-43.

54. Wen F, Zong F, Cheng WB, Gao K, Luo BL, et al. (2010) [HIV and current syphilis prevalence and related factors among men who have sex with men in Guangzhou]. South China J Prev Med 36: 19-23.

55. Weng YQ, Bai Y (2009) [Surveillance on the high risk behaviors among 239 men who have sex with men]. Journal of Applied Preventive Medicine 15: 152-153.

56. Zhong F, Lin P, Xu H, Wang Y, Wang M, et al. (2009) Possible Increase in HIV and Syphilis Prevalence Among Men Who Have Sex with Men in Guangzhou, China: Results from a Respondent-Driven Sampling Survey. AIDS Behav.

57. Li Y, Chen JJ, Gu LP, Gao WL (2007) [Survey of HIV/SY Infection and KABP Related to AIDS among the MSM in Lanzhou]. Chinese Medical Science & Health 8: 1-3.

58. Yang SP, Wang SR, Wang JY, Wang RF, Ayeti, et al. (2008) [Investigation on AIDS knowledge and behaviors among men who have sex with men in Hami prefecture]. Endemic Disease Bulletin 23: 49-50.

59. Miao ZF, Li J, Lei LM, Han X, Zhang XP (2009) [Survey of AIDS-related Knowledge and Behavior in 312 MSM]. Journal of Ningxia Medical University 31: 761-762.

60. Qiao XW, Ma XZ, Li Y (2009) [Epidemiological analysis of HIV-VCT service among men who have sex with men in the city of Lanzhou in 2008]. Health Vocational Education 27: 104-105.

61. Zhang M, Wang XD, Yang Y (2009) [Prevalence oh HIV, anti-HCV, syphilis infection and AIDS knowledge among men who have sex with men (MSM) in Urumqi]. Chin J Public Health 25: 1075-1076.

62. Zhang Y, Liu JW, Ni MJ, Dong YH (2008) [Laboratory Study on Detective Results of Male Homosexuality Population in Urumqi, Xinjiang]. Endemic Disease Bulletin 23: 26-28.

63. Lu CG, Yuan F, Shi ZH, Yang JZ, Li XY, et al. (2006) [HIV infection survey among MSM]. Chin J Public Health 2: 1320-1321.

64. Lu CG, Yuan F, Shi Z, Yang JZ, Li XY, et al. (2006) [The study of HIV infection and KABP about AIDS among the MSM in Guiyang city]. Guizhou Medical Journal 30: 202-204.

65. Feng L, Ding X, Lu R, Liu J, Sy A, et al. (2009) High HIV prevalence detected in 2006 and 2007 among men who have sex with men in China's largest municipality: an alarming epidemic in Chongqing, China. J Acquir Immune Defic Syndr 52: 79-85.

66. Zhou J, Zhu JJ, Bin H, Zhang L, Yao M, et al. (2008) [A survey of HIV/STD,HBV and HCV infections and risk behaviors among MSM in two central districts of Guiyang city]. Chinese Journal of AIDS & STD 14: 47-48, 51.

67. Ding XB, Feng LG, Xu J, Xu SM, Guo Xj, et al. (2010) [Study on the prevalence of HIV, Syphilis, HCV and HSV-II and its associated factors among 743 men who have sex with men in Chongqing]. Chinese Journal of Disease Control & Prevention 14: 227-231.

68. Feng Y, Wu Z, Detels R, Qin G, Liu L, et al. (2010) HIV/STD prevalence among men who have sex with men in Chengdu, China and associated risk factors for HIV infection. J Acquir Immune Defic Syndr 53 Suppl 1: S74-80.

69. Wang Y, Zhang HB, Gui ZG, Yang HW, Fan J, et al. (2008) [Analysis of behavior of MSM population and the biological monitoring result in Mianyang city]. Modern Preventive Medicine 35: 3780-3783, 3789.

70. Wang Y, Zhang HB, Zhang GG, Yang HW, Fan J, et al. (2009) [Biological Monitoring and Social Characteristics Investigation of MSM Group in Mianyang City]. Practical Preventive Medicine 16: 375-377.

71. Xiao Y, Ding X, Li C, Liu J, Sun J, et al. (2009) Prevalence and correlates of HIV and syphilis infections among men who have sex with men in Chongqing Municipality, China. Sex Transm Dis 36: 647-656.
